# Dynamics based alignment of proteins: an alternative approach to quantify dynamic similarity

**DOI:** 10.1186/1471-2105-11-188

**Published:** 2010-04-14

**Authors:** Márton Münz, Rune Lyngsø, Jotun Hein, Philip C Biggin

**Affiliations:** 1 Structural Bioinformatics and Computational Biochemistry, University of Oxford, South Parks Road, Oxford, OX1 3QU, UK; 2 Department of Statistics, University of Oxford, 1 South Parks Road, Oxford, OX1 3TG, UK; 3 Oxford Centre for Integrative Systems Biology, Department of Biochemistry, South Parks Road, Oxford, OX1 3QU, UK

## Abstract

**Background:**

The dynamic motions of many proteins are central to their function. It therefore follows that the dynamic requirements of a protein are evolutionary constrained. In order to assess and quantify this, one needs to compare the dynamic motions of different proteins. Comparing the dynamics of distinct proteins may also provide insight into how protein motions are modified by variations in sequence and, consequently, by structure. The optimal way of comparing complex molecular motions is, however, far from trivial. The majority of comparative molecular dynamics studies performed to date relied upon prior sequence or structural alignment to define which residues were equivalent in 3-dimensional space.

**Results:**

Here we discuss an alternative methodology for comparative molecular dynamics that does not require any prior alignment information. We show it is possible to align proteins based solely on their dynamics and that we can use these dynamics-based alignments to quantify the dynamic similarity of proteins. Our method was tested on 10 representative members of the PDZ domain family.

**Conclusions:**

As a result of creating pair-wise dynamics-based alignments of PDZ domains, we have found evolutionarily conserved patterns in their backbone dynamics. The dynamic similarity of PDZ domains is highly correlated with their structural similarity as calculated with Dali. However, significant differences in their dynamics can be detected indicating that sequence has a more refined role to play in protein dynamics than just dictating the overall fold. We suggest that the method should be generally applicable.

## Background

It is well established that conformational flexibility plays a key role in the biochemical functions of proteins [1,2]. Protein motions of functional importance range from fast (sub-nanosecond) atomic fluctuations to slow (microsecond upward), large-scale conformational rearrangements [3,4]. Several studies have managed to relate internal protein motions to biochemical functions [5,6], and in particular the characterization and prediction of large-scale conformational changes via the use of normal modes [7] and elastic-network models [8,9] has been quite successful. However, there are many signalling molecules (for example PDZ domains) where there is not a large-scale conformational change and yet somehow the information that a ligand has bound is communicated to a different region of the protein. Furthermore, it is not clear if slight variations in structure can lead to large variations in dynamics, or similar protein structures always have similar motions [9]. These problems require a more detailed picture of the underlying dynamics.

Molecular Dynamics (MD) simulations can be used effectively to explore the conformational energy landscape accessible to proteins [10,11] giving an insight into how protein dynamics relates back to sequence. Comparative MD studies address these questions by performing MD simulations of multiple proteins and comparing their dynamic trajectories. Previous comparative MD studies of proteins fall into two main categories. Studies in the first class compared the dynamics of the same protein simulated under different conditions [12-15]. In this case, the question is how the motion of the protein is altered by the new condition, for example the presence of a ligand. By contrast, the second class of studies compared the dynamics of different proteins simulated under the same condition [16-18] in order to pinpoint similarities and differences in functionally important movements.

If the dynamics of non-identical proteins is compared, a mapping between the different structures is often required. Therefore a common point of previous comparative MD studies of homologous proteins is that they use prior sequence or structure alignments to find residue equivalencies between the proteins. For example, to compare the fluctuation of different cold-active enzymes, Spiwok *et al*. [18] used structural alignmentto define equivalent residue pairs between the proteins. Papaleo *et al*. [17] performed MD simulations of different elastases to compare their molecular flexibility. Here the correspondences between residues of different proteins were derived from pairwise sequence alignments. Another example for alignment-guided comparison of protein dynamics is the work of Pang *et al*. [16] who simulated a series of proteins within the same fold family. To compare the fluctuations as well as the principal components of dynamics of only the structurally conserved residues across the set of proteins, they used structural alignment to define conserved positions. Alternatively to MD, simplified models called Gaussian Network Models were used to explore the common dynamics of the globin family [19] and the protease superfamily [20]. In these studies, however, comparative analysis of dynamics also relied on prior alignments of the proteins.

Nevertheless, if dynamically equivalent regions do not match to sequentially and structurally similar regions, alignments can mislead the comparison of protein motions. Recently, Zen et al [21,22] developed a method that takes a combined measure of spatial and dynamic consistency to derive an alignment on the fly that can be used to compare the proteins. We introduce here an alternative method to measure the similarity of backbone dynamics of proteins without the use of any prior alignment information. The method creates pairwise alignments of proteins based solely on their backbone motions without taking into account their sequence and structure. Scores of dynamics-based alignments are used to quantify the dynamic similarity of proteins. The proposed similarity measure can be applied to study the topology of protein dynamics space.

**Figure 1:**
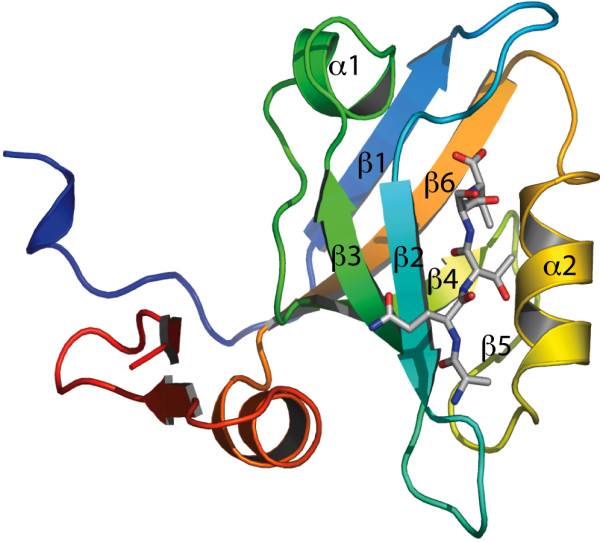
**The third PDZ domain (PDZ3) of Postsynaptic Density Protein-95 (PSD-95) in complex with the C-terminal peptide of CRIPT**. PDB code 1BE9.

The method is tested on members of the PDZ domain family. PDZ (post-synaptic density-95/discs large/zonula occludens-1) domains are common protein-protein interaction domains [23], most often binding the C-terminal of the ligand protein [24] (see Fig. 1). PDZ domains play an important role in organizing signalling complexes and ion channels [25,26]. The biophysical aspects of folding and binding reactions of PDZ domains has been intensively studied [27], as well as their ligand preferences [28,29] and the background of binding specificity against a wide range of ligands [30]. Due to their biological importance, PDZ domains are found in a large number of species: bacteria, yeast, plants, invertebrates and vertebrates [31]. The dynamics (collective normal modes) of PDZ domains in relation to functional properties was studied on a coarse-grained model [32]. Recently, Law *et al*. [33] have proven by NMR-relaxation experiments that PDZ domain side-chain dynamics is evolutionary conserved. In this paper we conclude that backbone dynamics of PDZ domains is also evolutionarily conserved. We first describe the development of the method and how it can be used to generate an alignment before demonstrating its application to PDZ domains.

## Results

### Dynamic Fingerprint Matrix

We introduce a novel way to characterize the backbone dynamics of proteins. The underlying idea is that in a moving protein each residue is fluctuating relative to all other residues, therefore a detailed description of motion should capture all inter-residue fluctuations. While static structures are often characterized by the matrix of inter-residue distances (i.e. the distance matrix), this representation is not applicable for a moving protein in which inter-residue distances are constantly changing. It is possible, however, to characterize the relative motion of any two residues by the distribution of their distance over time. We measure the extent to which residue *i *and residue *j *are fluctuating relative to each other by(1)

where *F*_*ij *_is calculated as the standard deviation of the distribution of *D*_*ij *_(distance of the two residues) in the whole conformational ensemble generated by MD simulation. In this initial investigation we only consider distances between Cα atoms, but the technique can easily be extended to a more detailed description of each amino acid residue. The standard deviation of the distance distribution reflects how much the two residues fluctuate relative to each other. *F*_*ij *_values are calculated for each residue pair and are collected into a matrix denoted by *F*, which we will refer to as the Dynamic Fingerprint Matrix (DFM). Similarly to a distance matrix that characterizes a single conformation, a dynamic fingerprint matrix characterizes an ensemble of conformations. (The relationship of DFMs and correlation matrices is discussed in Additional file 1.)

### Comparing DFMs using prior alignment

Where a prior alignment is known, the comparison of DFMs is straightforward. Given a pairwise sequence alignment of protein A and B, let α and β be the index vectors of the aligned residues of sequence A and B, respectively. That is, the *k*^th ^match column, (i.e. columns not containing a gap in the alignment) aligns residue α *(k) *of protein A with residue β *(k) *of protein B. Thus each pairwise alignment can be characterized by an (α,β) pair. Let F^A ^and F^B ^be the DFMs of the two proteins. The *(α,β) *alignment define a submatrix of size of |α|×|α| of both DFMs. The *(i, j) *entries of the two submatrices are given by(2)

Note that the two submatrices describe the pairwise fluctuations of the aligned residues only. The (*i, j*) entries of the two submatrices are considered equivalent as they describe the fluctuations of equivalent pairs of residues.

We define the dynamic similarity score of protein A and B based on a prior (α,β) alignment as:(3)

where each pair of equivalent matrix entries are compared one-by-one and their contribution to the overall score is given by(4)

where(5)

is the relative difference of the two equivalent matrix entries.

*s(i, j) *is an S-shaped logistic function that assigns positive score (*s*_+_) to highly similar matrix entries and negative score (s_-_) to highly dissimilar entries (Additional file 1: Fig. S1). An user-adjustable cut-off parameter, *t*, defines the critical *d(i, j) *over which *s(i, j) *turns negative. The relationship between *t *and parameter *ω *of *s(i, j) *is discussed in Additional file 1 as well as the choice of parameter values. The key difference from using a discrete threshold is that the parameter λ can be tuned to set the steepness of the S-shaped function to make *s(i, j) *less dependent on the cut-off parameter *t*. Since *s(i, j) *is associated with a match column pair in the alignment, it will be referred to as the Pairwise Match Score (PMS) of columns *i *and *j*.

### Comparing DFMs without prior alignment

In the previous section we have introduced how to compare the DFMs of two proteins using a prior sequence/structural alignment. Our main goal, however, is to find the optimal alignment of two proteins based on solely their DFMs. Note that it is the opposite strategy of previous comparative MD studies which relied upon prior alignments. We aim to find the *(α,β) *pair corresponding to the maximal similarity score. Let (α*, β*) be the pair of index vectors for which *S*^*AB*^*(α, β) *is maximal. *S*^*AB*^(α*, β*) is then called the dynamic similarity score of protein A and B and is simply denoted by *S*^*AB*^. The sequence alignment problem is hereby transformed into a matrix alignment problem. Structural alignment algorithms Dali [34] and MatAlign [35] aim to solve the same question when aligning distance matrices. The search space of (α,β) pairs is exponentially large and the global optimization problem is in fact NP-hard. In this case to find the maximum score *S*^*AB *^we employ a simulated annealing protocol (see Methods).

### Single Match Score (SMS)

Although the PMS scores concerning a given match column depend on the other match columns in the alignment, it is useful to compare the total contributions of each individual column to the alignment score. For match column *i*, the sum of PMS scores with respect to all other match columns will be referred to as the Single Match Score (SMS):(6)

In other words, the SMS of a match column is the score by which the total alignment score decreases in case of removing that match from the alignment. Matches of negative SMS values are beneficial to remove in optimizing the alignment. Hence the optimal dynamics-based alignment contains only positions of non-negative SMS values. Either studying a prior (sequence/structural) or a dynamics-based alignment, the SMS-profile represents our confidence in each aligned pairs of residues.

### Analysis of the motion of PSD-95 PDZ3

**Figure 2:**
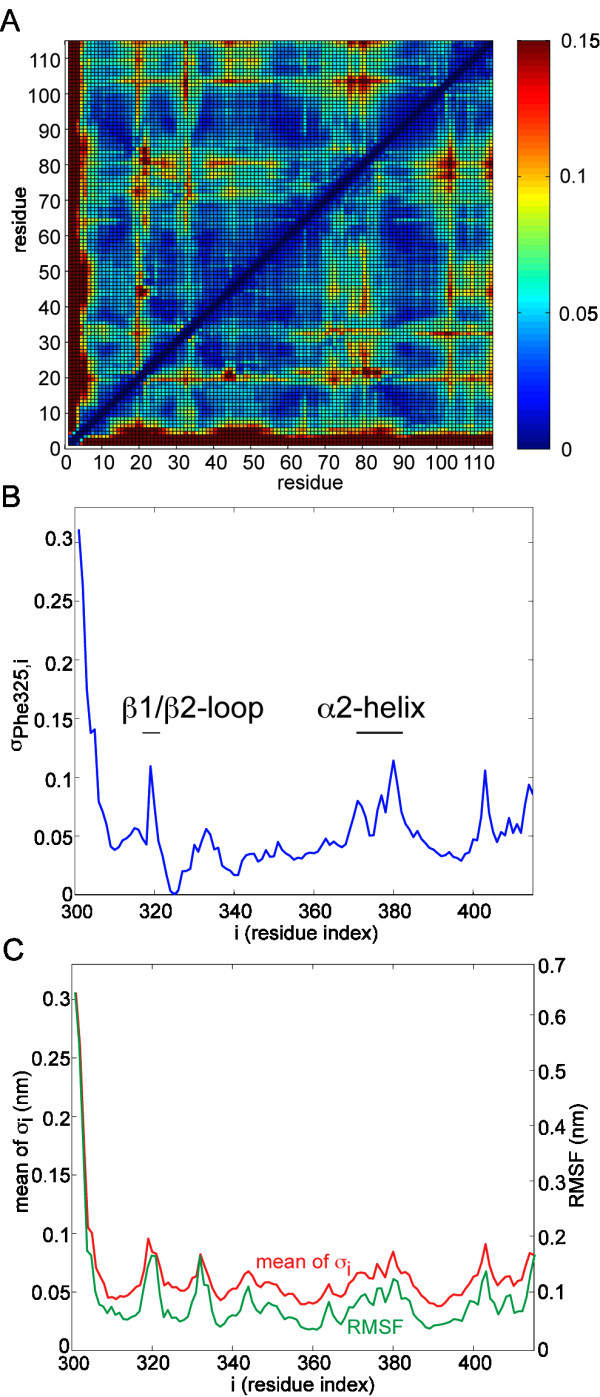
**Analysis of the DFM of PSD-95 PDZ3**. (A) shows the dynamic fingerprint matrix (DFM). Regions where the distance fluctuation gives a high standard deviation, σ, are indicated as red. Low σ values are indicated as blue. (B) Example dynamic 'profile' for residue 25 (F325 of PDZ3 of PSD-95). (C) Average fluctuation profile (the mean value of σ for residue i) compared to the RMSF profile.

Before we discuss the comparative analysis, we first demonstrate that the DFM protocol is appropriate by characterizing the dynamics of the third PDZ domain (PDZ3) of PSD-95 (Postsynaptic Density Protein 95) from Rattus norvegicus. PSD-95 plays an important role in controlling synaptic strength and plasticity in the central nervous system [36]. The 110-residue-long PDZ3 is the most well studied PDZ domain [27] which has a canonical PDZ-domain structure consisting of six β-strands (β1-β6) and two α-helices (α1 and α2). The peptide-binding groove is located between the β2-strand and α2-helix (see Fig. 1). As described in Methods, we used a 20 ns MD trajectory to calculate the Dynamic Fingerprint Matrix (DFM) of PDZ3 (see Fig. 2A). Simple analysis of the DFM revealed that the most dynamic part of the domain is the α2 helix (His372-Ala382). This observation is in accordance with De Los Rios *et al *[32] who performed Normal Mode Analysis of a Gaussian Network Model of PDZ3. As the α2-helix and the β2-strand form the binding pocket it seems likely that the considerable relative motion of these two structural components may be related to the capacity to bind ligands.

To see how a particular residue fluctuates relative to all other residues, we examined individual rows of the DFM. We will refer to the *k*^*th *^row of the DFM as the dynamic profile of residue *k*. The dynamic profile of Phe325, for example, shows us that it is the β1/β2 loop and the α2-helix which fluctuates the most relative to this residue (see Fig. 2B). Phe325 is at the N-terminal end of the β2-strand, right next to the β1/β2 (L1) loop that interacts with the carboxylate terminal of the bound peptide. Therefore, the relative motion of Phe325 and the α2-helix at the other side of the binding pocket along with the L1 loop may reflect the manner in which peptide binding occurs. A further example profile from a residue situated within an alpha helix (Asp 348) is shown Additional file 1.

We have calculated the mean value of each rows of the DFM which we will call the average fluctuation profile (Fig. 2C). Atomic fluctuations are often characterized by the RMSF (root mean square fluctuation). It can be seen that the RMSF profile is very similar to the average fluctuation profile (Fig. 2C). There was 0.94 correlation between the average fluctuation profile and the RMSF profile. We conclude that a DFM contains the same information as a standard RMSF plot, however, by describing relative inter-residue fluctuations, it gives us a more detailed representation of protein flexibility. More importantly, it does away with the dependency on a single "native" reference structure for defining fluctuations and is simple to compute.

### Dynamics-based alignments of PDZ domains

**Figure 3:**
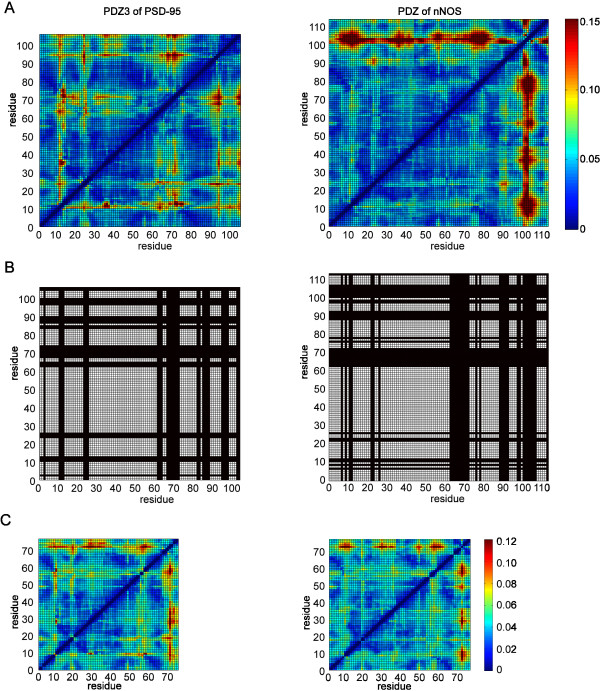
**(A) Comparison of DFMs for PDZ3 of PSD-95 with the PDZ domain of nNOS**. (B) Identification of similar submatrices, containing 77 residues in this case, from the DFMs. (C) Collapsed DFMs highlighting the similarities in these proteins.

We have thus far demonstrated that the DFM methodology can be a useful way to analyse protein motions, but the power of the approach is that it enables us to compare the dynamics of two or more different proteins. Furthermore this information can be used to derive an alignment. To illustrate this we selected 10 PDZ domains from a range of organisms (see Table 1) and ran 20 ns explicit MD simulations (see Methods). We then calculated the DFMs for each protein and created dynamics-based alignments of each pairs of proteins using the matrix alignment algorithm described in Methods. Fig. 3. presents an example: the alignment of PDZ3 of PSD-95 and the PDZ domain of neuronal nitric oxide synthase (nNOS). The alignment does not require any prior sequence or structural information; the two DFMs are the only inputs of the algorithm (Fig. 3A). The most similar submatrix pair found by the algorithm have 77 × 77 entries which are highlighted (in white) in Fig. 3B. The optimal submatrix pair corresponds to a pairwise alignment consisting of 77 aligned residues. Removing the rows and columns of the DFMs that correspond to gaps in the alignment, the remaining matrices will be referred to as the 'collapsed DFMs' (which are identical to the submatrices identified). Although one cannot see notable similarity between the original DFMs, the collapsed DFMs appear to be visually similar patterns (Fig. 3C).

**Table 1:** Representative members of the PDZ domain family used in this study.

Protein containing PDZ domain	PDB entry	Resolution (Å)	Source organism
nNOS	1qau	1.25	*Rattus norvegicus*
InaD	1ihj	1.8	*Drosophila melanogaster*
PSD-95 (PDZ3)	1bfe/1be9	2.3/1.82	*Rattus norvegicus*
tricorn protease	1k32	2.0	*Thermoplasma acidophilum*
GRIP2 (PDZ4)	1x5r	NMR structure	*Homo sapiens*
Rv0983	1y8t	2.0	*Mycobacterium tuberculosis h37rv*
PhotosystemII D1 Protease	1fc6	1.8	*Scenedesmus obliquus*
Alpha-1 Syntrophin	1qav	1.9	*Mus musculus*
EpsC	2i6v	1.63	*Vibrio cholerae*
DVL2	2f0a	1.8	*Xenopus laevis*

**Figure 4:**
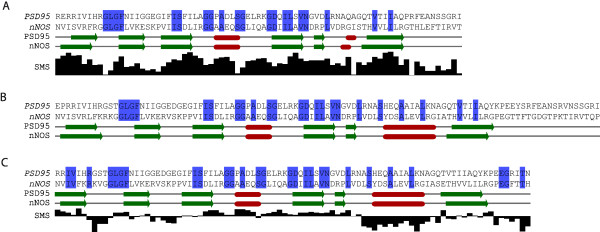
**The resulting alignment that can be derived from the collapsed DFMs (A) is compared to the alignments derived from the Dali (B) and Needleman-Wunsch (NW) algorithms (C)**. Identical pairs are indicated by blue boxes. The single match score (SMS) is depicted underneath the DFM and NW alignments. It can be seen that the region corresponding to the second alpha helix in the NW alignment gives negative SMS values indicating that the dynamic similarity is not preserved in this region.

The derived dynamics-based alignment was compared to a structural alignment created by pairwise DaliLite [37] and a pairwise sequence alignment created by the Needleman-Wunsch algorithm [38] using EMBOSS-Align [39]. Fig. 4. presents the three alignments annotated by the secondary structure elements of the canonical PDZ-domain fold (i.e. six beta-strands, β1 to β6, and two alpha-helices, α1 and α2). For the DFM-alignment and the sequence alignment, the SMS score of each column is also presented, reflecting our confidence in individual aligned positions.

As shown by Fig 4, equivalent secondary structure elements of the two proteins align very well in the dynamics-based alignment, suggesting that dynamics, just like sequence and structure, may contain enough information to match proteins at the secondary structure level. Moreover, the DFM-based alignment includes 20 pairs of identical residues, out of which 18 and 20 are also present in the Needleman-Wunsch and Dali alignments, respectively. Despite all these similarities, however, a striking difference can be seen between the DFM-based alignment and the sequence/structural alignments. The second alpha-helix (α2), included both in the Needleman-Wunsch and Dali alignments, is almost completely missing from the DFM-alignment, indicating that, although conserved at the sequence and structure level, this helix has different dynamics in the two proteins. Characterized above, the α2-helix has high mobility in the PDZ3 of PSD-95 unlike in the PDZ of nNOS, that makes the two regions dynamically non-alignable. This is a clear example, when the dynamics-based alignment gives similar information as sequence and structural alignments, but at the same time, it provides new insights into the properties of proteins, that cannot be detected through standard alignment methods.

### Analysing SMS-profiles

Since it was optimized by the matrix alignment algorithm, the DFM-alignment includes only matches of positive SMS values as shown by Fig. 4A. The SMS-profile has its peaks within β-strands but drops at certain match columns (e.g. in the β1/β2 and β2/β3 loops and at the C-terminal end of α1-helix). This suggests that β-strands of the domains have minor fluctuations that makes them easier to align than the other regions of the proteins.

As we discussed before, the dynamic similarity of proteins can also be measured based on a prior (sequence or structural) alignment. In this case, the motion of the subsets of residues defined by the prior alignment is compared. To test this option, we used the Needleman-Wunsch alignment as a prior alignment, which resulted in a dynamic similarity score of -132.8. The optimal similarity score found for this example is 1307.3, and the extreme non-optimality of the sequence based alignment score illustrates that conserved sequence positions can match dynamically dissimilar subsets of residues. Accordingly, 46 per cent of the columns of the collapsed Needleman-Wunsch alignment have negative SMS. The less-matching region (a continuous block of negative SMS values) appears to be the α2-helix, explaining why this region is excluded from the optimized DFM-alignment.

### Dynamics-space of PDZ domains

The dynamic similarity score of the PDZ domain of nNOS and PDZ3 of PSD-95 is S = 1307.3, which was converted to a *p*-value of 9·10^-11 ^using the significance analysis framework described in Methods. Likewise the alignment algorithm has found significant dynamic similarities between other pairs of PDZ domains. The p-values are summarized in Table 2 and can be shown as a dynamic similarity graph Additional file 1: Fig S4A, in which the different proteins are represented by the nodes of the graph, and two proteins are connected if they have significantly similar (*p*-value < 0.05) dynamics.

**Table 2:** Dynamic similarity *p *values for the 10 PDZ domains.

	1qau	1ihj	1be9	1k32	1x5r	1y8t	1fc6	1qav	2i6v	2f0a
1qau										
1ihj	4·10^-4^									
1be9	9·10^-11^	0.34								
1k32	0.62	0.76	0.74							
1x5r	0.32	0.32	0.60	0.39						
1y8t	0.61	0.47	0.58	0.62	0.36					
1fc6	0.08	8·10^-3^	0.78	0.76	0.65	0.19				
1qav	1·10^-8^	2·10^-3^	1·10^-5^	0.66	0.14	0.07	0.17			
2i6v	0.68	0.78	0.58	0.70	0.71	0.55	0.81	0.59		
2f0a	1·10^-4^	3·10^-6^	0.27	0.30	8·10^-4^	0.14	0.47	6·10^-6^	0.72	

Those that are significant (at the *p *< 0.05) are highlighted.

It is interesting to note that the dynamic similarity shows differences between different structures. We can see a cluster of five proteins (1be9, 1qau, 1qav, 1ihj and 2f0a) that are better-connected in the dynamic similarity graph. Out of the 10 possible links between these structures 8 are present in the graph. Two additional links are found between 2f0a/1x5r and 1ihj/1fc6. Three structures (2i6v, 1k32 and 1y8t), however, do not have significant dynamic similarity with any other structures. Looking for structural explanations for these differences, we examined the pairwise Dali Z-scores between the 10 domains (summarized in Additional file 1: Table S1 and shown as a Dali Z-score graph in Additional file 1: Fig. S4B). In this second graph, two nodes are connected if their Dali Z-score is more than 8.5 (a threshold selected empirically). As expected, each protein pair has significant structural similarity (all Dali Z-scores are over 3.5), but a subset of structures are more similar to each other than to the others. A cluster of six structures (1be9, 1qau, 1qav, 1ihj, 2f0a and 1x5r) appears to be fully connected in the graph, while two additional links are found between 1fc6/1y8t and 1ihj/1fc6. Two domains (1k32 and 2i6v) are not linked to any other structures.

The almost perfect overlap (with the only exception of 1x5r) between the well-connected clusters in the two graphs suggests a topology-preserving mapping between the structure space and dynamics space of PDZ domains. There appears to be a strong correlation (0.82) between the raw dynamic similarity scores and Dali Z-scores considering all 45 protein pairs (Additional file 1: Fig. S5), and a weaker but still strong correlation (0.63) considering only the 35 protein pairs having non-significant dynamic similarities.

Interestingly, all the 6 proteins in the fully connected cluster of the Dali graph (5 of which are well-connected in the dynamics graph too) are from multicellular organisms (metazoa), while the other 4 proteins are from unicellular species. The structural difference between PDZ domains from simple and complex organisms is well-known. First recognized by *Liao et al*. [40] and exemplified by other authors [41,42], PDZ domains of bacterial and plant origin have a circularly permuted fold compared to the canonical PDZ domain fold found in metazoa. Despite their considerably different architecture, non-metazoan PDZ domains have very similar overall tertiary structure to metazoan PDZ domains. This is indeed reflected by Additional file 1: Table S1 which shows that metazoan and non-metazoan PDZ domains are significantly similar structures (Dali Z-scores above 2). On the other hand, the fact that the metazoan structures form a distinct cluster in the Dali graph shows the difference between the canonical and the circularly permuted fold. Putting it all together, our data suggests that the essential structural difference between PDZ domains of metazoan and non-metazoan origin is also reflected by the dissimilarity of their dynamics. Metazoan PDZ domains appear to be structurally and dynamically more conserved. However, even within the cluster of the metazoan proteins, there are significant differences in dynamics (on this timescale) that can be quantified.

### Robustness of DFMs

MD simulations are subject to sampling problems. In order to assess whether the simulations have run long enough to provide a reasonable picture of the dynamics we examined the convergence of the DFM patterns. We ran five 20 ns simulations of the same protein (PSD-95 PDZ3) using different random seeds for the initial atomic velocities. The similarities of each pairs of DFMs resulted from the different MD runs were measured by the matrix alignment algorithm. Naturally, the five DFMs were not perfectly the same, but the similarity between each pair was highly significant (see Table 3). Most importantly, comparing different simulations of the same protein results in much higher similarity scores, than the comparison of different PDZ domains. These results lead us to the conclusion that the sampling in 20 ns simulations can be sufficient to provide robust DFM patterns for a comparative analysis. Clearly, simulating the proteins for longer period of time further improves the convergence of DFMs.

**Table 3:** Dynamic similarity scores of five trajectories of 1be9 with different initial seed velocities.

	Run 1	Run 2	Run 3	Run 4	Run 5
**Run 1**					
**Run 2**	2363.3				
**Run 3**	3223.7	3029.8			
**Run 4**	2283.9	1589.4	1715.7		
**Run 5**	2693.1	2221.1	2692.8	2244.1	

All pairs are highly significant (p-value ≈ 0).

## Conclusions

We have demonstrated a novel methodology for comparing the backbone dynamics of proteins simulated by Molecular Dynamics simulations and for deriving an alignment that is based solely on the underlying dynamics of the system within a particular timescale; in this case 20 ns. We have selected that timescale for practical reasons, but it is effectively arbitrary as we were mainly interested in seeing if we could quantify the similarity in a meaningful way. The method should work just as well for longer timescales. Of course, the most useful timescale is the one which proteins exhibit their function. The comparison of our dynamic similarity score with the Dali score demonstrates structure and dynamics are indeed correlated, but at a level (R = 0.82) that still allows for significant differences in dynamics to be apparent. This may explain why methods such as elastic network models seem to work for predicting large-scale conformational changes, but that the detailed differences between protein motions can still be significant. With respect to the PDZ domains we have shown that the majority of motions within the protein are similar, but additionally that we can detect differences (located at the binding interface) that are significantly different and could not be detected by the usual sequence or structure based alignment methods. We have developed a method for measuring dynamic similarity between proteins with a simple algorithm. As the method is capable of detecting precise differences in the dynamics between structures it could also be used to assess the influence of ligand-binding on the dynamics of protein structure. We are currently exploring that aspect as well as developing further improvements on the algorithm.

## Methods

### Molecular Dynamics Simulations

We carried out all-atom MD simulations of 10 representative members of the PDZ domain family (Table 1) in explicit water and with Na^+ ^and Cl^- ^ions to a concentration of 150 mM. After 200 ps of restrained MD, 20 ns of unrestrained MD was performed with the GROMACS software package [43,44] using the OPLS force field [45] in an NPT ensemble (see Additional file 1 for full details). Snapshots from the trajectories were saved every 5 ps for analysis.

#### Matrix Alignment Algorithm

To find a good approximation for the global maximum of *S*^*AB*^*(α,β) *in a reasonable time, we have developed a heuristics approach based on the multiple restart Simulated Annealing (SA) method. The algorithm performs an MCMC (Markov chain Monte Carlo) search in the space of *(α,β) *pairs. The Markov chain starts from a random initial alignment, and in each step the alignment is modified by inserting or removing one residue pair. We use the Metropolis acceptance criterion [46] to decide the next state of the chain. The parameter called 'temperature' which controls the acceptance probability is gradually reduced according to an exponential decay annealing schedule, leading to the convergence to a high-scoring and potentially optimal alignment. We let the Markov chain explore the search space at a given constant temperature: the chain has to go through a minimal number of accepted steps before the temperature is further reduced. The initial temperature is calibrated using the method proposed by Johnson et al. [47]. The whole SA procedure terminates when the acceptance ratio goes below a critical value. To overcome the stochastic nature of SA and the possible existence of local optima, the process is restarted for a number of times from random initial states and the best result of the multiple runs is selected as the final output of the algorithm.

#### Significance Analysis

To assess the statistical significance of dynamic similarity scores between PDZ domains, we have performed 20 ns MD simulations of 12 evolutionarily and functionally unrelated proteins of different sizes referred to as the Reference Set (Additional file 1: Table S2). Reference proteins were aligned using our dynamic fingerprint alignment algorithm to measure the background distribution of similarity scores. Using the background distribution, the significance of dynamic similarity of any two proteins can be expressed by the p-value of their similarity score.

Since the optimal alignment score of two proteins is the maximum of the scores of their possible alignments, it follows a type I Extreme Value Distribution, as shown in Additional file 1: Fig. S2. The background score distribution was found to be dependent on the lengths of sequences (i.e. the size of input DFMs). To capture the size dependency of the background distribution, we approximated the *μ (L) *and *σ(L) *functions, where *μ *is the location parameter, *σ *is the scale parameter of the Extreme Value Distribution and *L = L*_*A*_*L*_*B *_is the product of the two sequence lengths.

To measure *μ (L) *and *σ(L) *in a wider interval, not only the full DFMs but their submatrices of different sizes were also aligned. A total number of 2970 dynamic alignments were performed. Regression lines were fitted to the measured points of *μ (L) *and *σ(L) *and are used to approximate the parameters of the background distribution (Additional file 1: Fig. S3). The *p*-value of a dynamic alignment of protein A and B is therefore given by(7)

where *L *= *L*_*A*_*L*_*B*_, and *L*_*A *_and *L*_*B *_are the lengths of protein A and B, respectively. (For a more detailed explanation of the applied significance analysis framework, see Additional file 1.) We use a significance threshold of *p *= 0.05 to detect significant dynamic similarities between proteins.

## Abbreviations

PDZ: post-synaptic density-95/discs large/zonula occludens-1; DFM: dynamics fingerprint matrix.

## Authors' contributions

JH and PCB designed the research. MM performed the research. MM, RL and PCB analyzed the data. MM, RL and PCB wrote the paper. All authors read and approved the final manuscript.

## Supplementary Material

Additional file 1 **Supplementary Information**. Contains detailed information about the actual algorithms employed, data sets used, and extended interpretation of the data presented in the main manuscript. Click here for file
