# Supplementary material for: Dynamics based alignment of proteins: an alternative approach to quantify dynamic similarity
Source: BMC Bioinformatics. 2010 Apr 14;11:188. doi: 10.1186/1471-2105-11-188 (PMC2868010; doi:10.1186/1471-2105-11-188)
Supplement: Additional file 1 — Supplementary Information. Contains detailed information about the actual algorithms employed, data sets used, and extended interpretation of the data presented in the main manuscript. [file 1471-2105-11-188-S1.PDF]

Supplementary Information for Münz et al.

1. Pairwise Match Score (PMS)

Let *i* and *j* be two match columns of the *(α,β)* alignment, i.e. residue *α(i)* is aligned to residue *β(i)* and residue *α(j)* is aligned to residue *β(j)*. The Pairwise Match Score of columns *i* and *j* is the contribution of the two aligned positions to the total similarity score and is calculated by

$$s(i,j) = \frac{s_+ - s_-}{1 + e^{\lambda(d(i,j)-\omega)}} + s_-$$

where

$$d(i,j) = \frac{\left| F^A_{\alpha(i)\alpha(j)} - F^B_{\beta(i)\beta(j)} \right|}{\left(F^A_{\alpha(i)\alpha(j)} + F^B_{\beta(i)\beta(j)} \right) / 2}$$

is the relative difference of the two equivalent DFM matrix entries, $F^A_{\alpha(i)\alpha(j)}$ and $F^B_{\beta(i)\beta(j)}$.

s(i,j) is an S-shaped logistic function, which has four free parameters, s_+, s_-, λ and ω. The first two parameters define the maximum and minimum of *s(i,j)*. For highly similar matrix entries *s(i,j)* is close to s_+, while for highly dissimilar entries *s(i,j)* is close to s_-. Changing the ratio of s_+ and s_- has a similar effect to changing the ratio of match and mismatch scores in a standard sequence alignment algorithm. Using high s_-/s_+ ratio, the matrix alignment algorithm can be forced to exclude match columns that align non-similar matrix entries with other match columns.

The parameter ω of the logistic function determines the zero level of the Pairwise Match Score. Let t be defined as a cut-off value at which *s(i,j)* turns from positive to negative. If the relative difference of two matrix entries is smaller than the cut-off parameter t, their PMS increase the total alignment score, otherwise it has a negative contribution. The transformation between the user-adjustable parameter t and parameter ω of s(i,j) is given by

$$\omega = t - \frac{1}{\lambda} \ln\left(- \frac{s_+}{s_-} \right)$$

The fourth parameter, λ, can be used to set the steepness of the logistic function (the transition between s_+ and s_-) to make *s(i,j)* less dependent on the choice of the cut-off parameter t.

An example for the S-shaped *s(i,j)* function is shown in Fig. S1, with the parameter values of

$s_+=1$, $s_-=-1$, $\lambda=100$ and $t=0.2$

The above-listed parameter values of the Pairwise Match Score were selected empirically by trying to increase the discriminative power of the scoring function as much as possible. That

is, we wanted to find parameter values which result in a large difference between similarity scores of PDZ domains and similarity scores of random proteins. For example, if the value of the cut-off parameter t is too small, it may give so strict requirement on the similarity of DFM entries that is rarely satisfied regardless we are aligning PDZ domains or random proteins. On the other hand, too large values of parameter t result in equally good scoring alignments of random proteins and PDZ domains. The choice of t=0.2, which proved to be reasonably good, means that two equivalent DFM matrix entries can differ by a maximum of 20 per cent (relative to their mean) in order to give a positive contribution to the alignment score. The rationality behind using $s_+=1$, $s_-=-1$ is that we wanted to let the highly similar and highly dissimilar matrix entry pairs have the same weight in the total alignment score. It is analogous to the case when matches and mismatches are equally weighted in standard sequence alignment algorithms. When the s_-/s_+ ratio is increased, the resulting dynamics-based alignments typically contain less match columns, but the number of columns corresponding to negative PMS values is also reduced.

2. Details of the Matrix Alignment Algorithm

The goal of the algorithm is to find the (α^*, β^*) index vector pair for which $S^{AB}(\alpha^*, \beta^*)$ is the global maximum of the $S^{AB}(\alpha, \beta)$ similarity function. For convenience, we denote $x=(\alpha, \beta)$ to represent a single element of the search space. Note that although we address a global *maximization* problem, the algorithm presented here aims the global *minimization* of the $E(x)= -S^{AB}(x)$ function, which is equivalent to the original problem. E(x) will be referred to as the energy of state x. The inputs of the algorithm are the two dynamic fingerprint matrices (DFMs), while the output is the $x^*=(\alpha^*, \beta^*)$ best alignment encountered.

The main steps of the algorithm

Here we present the optimization algorithm with explanations of each steps in the following section.

```
x_0 := randomAlignment()
x_best:=x_0
T_0 := estimateInitialTemperature()
j := 0
while (acceptance_ratio > AR_min) {
i := 0
accepted_steps := 0
  while (acceptedsteps < AS_min) {
      x' := Propose(x_i)
      p := min{1,exp(E(x)-E(x') /T_j)
      r := U[0,1]
      if (r<p) {
        x_i+1 := x'
        accepted_steps = accepted_steps+1
        }
      otherwise   x_i+1:= x_i
      i := i+1
      acceptance_ratio := accepted_steps/i
      if E(x_i)<E(x_best)  x_best:=x_i
```

20 }
21 T_{j+1}: = T_j/1.2
22 j: = j+1
23 }
24 Output: x_{best}

Description

The algorithm uses a Markov chain Monte Carlo (MCMC) method to perform stochastic optimization. In Step 1, the initial state of the Markov chain is set to a randomly generated $x_0=(\alpha_0, \beta_0)$ alignment.
According to the Simulated Annealing (SA) heuristics, the temperature (T) of the Markov chain is gradually reduced during the search. The double cycles (Step 5 and 8) control the run of SA. In the inner cycle (Step 8 to 20), a Markov chain of constant temperature is created. A proposal function (see in the next section) is used in Step 9 to randomly modify the current state of the Markov chain. The probability of accepting the modified state (x') as the next state of the chain is calculated in Step 10 according to the Metropolis acceptance criterion (Metropolis et al. 1953). If a random number (r) drawn from uniform distribution (Step 11) is lower than the calculated probability (p), the next state of the Markov chain will be the modified state, otherwise it will be the previous state (Step 12 to 16). The best state (with the lowest E) found so far is saved to x_{best}, which is initialized in Step 2. As the Markov chain grows, it is always checked in Step 19 if the current state of the chain is better than the saved x_{best} state. If so, x_{best} is updated. The number of accepted steps (in which x_{i+1} := x') is counted by Step 14. The inner cycle does not stop until the number of accepted steps exceeds a pre-defined threshold (AS_{min}), ensuring that the chain appropriately explores the accessible states (Step 8).
Note that in the Metropolis criterion, the probability of accepting less-optimal states depends on the temperature of the Markov chain. When the inner cycle terminates, the temperature parameter is reduced according to an exponential decay annealing schedule (Step 21). The whole process restarts again: a Markov chain of the new constant temperature is being generated in the inner cycle.
Therefore the outer cycle (Step 5 to 23) controls the annealing process, in which the temperatures of the consecutive Markov chains are getting lower and lower. The outer cycle terminates (Step 5) if the acceptance ratio calculated in Step 18 goes below pre-defined a cutoff (AR_{min}). The acceptance ratio is calculated as the number of accepted steps (accepted_steps) divided by the total number of steps (i). Note that both counters are set to zero when the inner cycle is restarted (Step 6 and 7). Low acceptance ratio indicates that the Markov chain is trapped into one or few states due to the low temperature.
The initial value of the temperature parameter (T_0) is calculated in Step 3 using the method proposed by Johnson et al. (1989, 1991) In this, a short trial MCMC run is performed and the optimal initial temperature is estimated from the average energy (score) differences.
When the outer cycle terminates, the algorithm ends. The output is the best state found (x_{best}).

The proposal function

As shown in the previous section, the role of the proposal function Propose(x) is to introduce a random modification into the current state of the Markov chain. Let the current state of the

chain be x=(α, β) and let x'=(α', β') be the modified state, where x'=Propose(x). The proposal function simply adds or removes an index pair to or from the α, β index vectors. In case a new index pair (i_A and i_B) is added, the resulting index vectors are given by

$$\alpha' = \alpha \cup \{i_A\} \quad \text{and} \quad \beta' = \beta \cup \{i_B\}$$

In case an existing index pair (i_A and i_B) is removed, the modified index vectors are

$$\alpha' = \alpha \setminus \{i_A\} \quad \text{and} \quad \beta' = \beta \setminus \{i_B\}$$

In each step the probability of adding a new index pair is 0.6, while the probability of removing an old index pair is 0.4. The proposal function first decides whether it adds or removes an index pair and then it randomly selects the index pair to be added or removed. While it is straightforward to select a removable index pair, selecting an insertable index pair is not trivial. To understand this, let (i_A^1,i_B^1), (i_A^2,i_B^2) and (i_A^3,i_B^3) be three aligned index pairs for which the order is $i_A^1 < i_A^2 < i_A^3$ and $i_B^1 < i_B^2 < i_B^3$. Let a randomly generated index pair be denoted by (i_A^*,i_B^*). In case $i_A^1 < i_A^* < i_A^2$ and $i_B^1 < i_B^* < i_B^2$, the (i_A^*,i_B^*) pair can be inserted into the alignment. Similarly, if $i_A^2 < i_A^* < i_A^3$ and $i_B^2 < i_B^* < i_B^3$, the (i_A^*,i_B^*) pair is also insertable. However, inserting the (i_A^*,i_B^*) pair would cause a conflict in the order of aligned residues in case $i_A^1 < i_A^* < i_A^2$ and $i_B^2 < i_B^* < i_B^3$. Consequently, not all randomly generated index pairs are insertable into the current state x. The proposal function therefore selects randomly only from the subset of insertable index pairs.

Random restarts

The method of Simulated Annealing does not guarantee to find the exact (global) maximum of the scoring function. In some cases, at low temperature the Markov chain is getting trapped by a local maximum. The more local maxima the scoring function has, the larger the chance for this quasiergodic behaviour which is also called the freezing problem. Because of the stochastic nature of the algorithm, its output is not always the same. Even if in some cases the algorithm succeeds to find the global maximum of the scoring function, in other cases it may fail. Therefore, in order to increase the reliability of the algorithm, the whole Simulated Annealing process can be restarted again and again from different random initial states. The number of restarts is an important parameter which be considered as a trade-off between the reliability and the runtime of the optimization.

Parameters of the algorithm

Besides the number of restarts, there are another two parameters that are able to improve the reliability of the algorithm at the cost of the runtime: AS_{min} and AR_{min}. By increasing the value of AS_{min}, one can help to achieve better convergence in each constant temperature sections of the Markov chain. Since the value of AR_{min} determines the point when the algorithm terminates, it should be low enough to let the whole Simulated Annealing process achieve convergence. Reducing AR_{min} increases the probability that SA will not be terminated too early. Another strategy, however, may be to set the parameters to get relatively short runtime and run multiple restarts. AS_{min}= 30000 and AR_{min}=0.05 were used in our experiments.

Making the algorithm faster

It is time consuming to re-calculate E(x') each time when the proposal function generates a new x' state. A much better strategy is to calculate only the $\Delta E(x) = E(x')-E(x)$ difference resulting from the modification of the previous state x. If a new index pair is added to state x by the proposal function, the energy difference is given by

$$\Delta E = -\sum_{k=1,k\neq k^*}^{|\alpha'|} s(k,k^*)$$

where $\alpha'(k^*)=i_A$ and $\beta'(k^*)=i_B$ are the inserted indices.
If an index pair is removed from state x by the proposal function, the energy difference is given by

$$\Delta E = \sum_{k=1,k\neq k^*}^{|\alpha|} s(k,k^*)$$

where $\alpha(k^*)=i_A$ and $\beta(k^*)=i_B$ are the removed indices.
The energy of the modified state is then calculated as $E(x')=E(x)+\Delta E(x)$. This strategy significantly speeds up the algorithm.

3. Significance Analysis

As discussed in Methods, raw S^{AB} similarity scores are converted into p-values in order to express the statistical significance of dynamic similarity. To calculate the p-values, we need the background score distribution, i.e. the distribution of scores resulting from aligning evolutionarily unrelated proteins. The background score distribution, however, may and does depend on the lengths of the two protein sequences (i.e. the sizes of the two DFMs). Therefore, when analysing the significance of dynamic similarity score between two proteins, one should always take into account the lengths of the sequences. Here we describe our strategy to measure the length-dependency of the background distribution.

45 distributions

Let $\Theta(L_A,L_B)$ be the background score distribution resulting from alignments of unrelated proteins of the length of L_A and L_B. The distribution is approximated for a set of different (L_A,L_B) pairs. Nine equally distributed points were selected in the [70,110] protein length interval: 70, 75, 80, 85, 90, 95, 100, 105 and 110. Using these points all different (L_A,L_B) pairs were generated. These are: *(70,70), (70,75), (70,80), (70,85), (70,90), (70,95), (70,100), (70,105), (70,110), (75,75), (75,80), (75,85), (75,90), (75,95), (75,100), (75,105), (75,110), (80,80), (80,85), (80,90), (80,95), (80,100), (80,105), (80,110), (85,85), (85,90), (85,95), (85,100), (85,105), (85,110), (90,90), (90,95), (90,100), (90,105), (90,110), (95,95), (95,100), (95,105), (95,110), (100,100), (100,105), (100,110), (105,105), (105,110)* and *(110,110).* (Note that only one of (x,y) and (y,x) is included in the list.) The $\Theta(L_A,L_B)$ distributions corresponding to the above listed 45 different length combinations were approximated.

66 protein pairs

Not only the full DFMs of the 12 reference simulations (Table S2), but their submatrices of any sizes can be used as inputs of the alignments. For example, if we want to align a protein of length L_A and a protein of length L_B, we can take a pair of reference proteins and use $L_A x L_A$ and $L_B x L_B$ submatrices of their DFMs.

The method of selecting a submatrix from an original DFM is the following. An integer (r) is randomly selected from the [0,N-S] interval, where N is the size of the DFM and S is the size of the future submatrix. The (i,j) entry of the submatrix is then defined as the (i+r,j+r) entry of the original DFM. In other words, we select a random subset of consecutive residues from the protein, and the resulting submatrix is the DFM describing the selected residues only.

As all the 12 reference DFMs are larger than 110x110, each DFMs can be used to extract input matrices for the 45 above-listed $\Theta(L_A,L_B)$ distributions. Since there are 66 different pairs of the 12 reference proteins, 66 alignments were performed for each (L_A,L_B) length combinations, in which the aligned $L_A x L_A$ and $L_B x L_B$ submatrices were taken from the 66 possible pairs of reference DFMs. As a result, each of the 45 $\Theta(L_A,L_B)$ distributions consists of 66 values: the dynamic similarity scores of 66 different alignments.

2970 alignments

Since the background distribution is studied for 45 different length combinations, and for each combination 66 alignments are performed, the total number of alignments carried out is 45x66=2970. All alignments were performed using the same parameter values (as described in the previous section).

Extreme Value Distribution

Since the optimal alignment score of two proteins is the maximum of the scores of their possible alignments, it follows a type I Extreme Value Distribution. To give an example, we show the measured $\Theta(80,80)$ distribution (histogram) in Fig. S2, that was generated by the method described above. However, to further-increase the accuracy of the distribution in this single example, not only one but ten independent alignments were performed for each reference protein pairs by repeating the random submatrix selection ten times. It therefore resulted in a distribution of 10x66 = 660 alignment scores. The extreme value distribution fitted to the 660 data points is also shown in Fig. S2.

Length-dependency of parameters

Since type I. Extreme Value Distribution has two parameters: the location parameter (μ) and the scale parameter (σ), we are only interested in the $\mu(L_A,L_B)$ and $\sigma(L_A,L_B)$ functions. To simplify the problem, we use a new variable $L=L_A \cdot L_B$ calculated as the product of the two sequence lengths. Hence the goal is to approximate the $\mu(L)$ and the $\sigma(L)$ functions. For each measured $\Theta(L_A,L_B)$ distributions, a type I. Extreme Value Distribution was fitted to the 66 data points. The Maximum Likelihood estimates of the location and scale parameters were then plotted against L. As presented in Fig. S3, linear functions fit well to the 45 data points in $\mu(L)$ and $\sigma(L)$.

As it is clear from Fig. S3, the background score distribution indeed depends on the lengths of the aligned proteins. Both the location (μ) and the scale (σ) parameters of the distribution increase with the size of the aligned DFMs. Consequently, if the length-dependency was not taken into account when calculating the p-values, the statistical significance of similarity could be over-estimated for large protein pairs.

The equations for the best fit lines are given by

$$\mu(L) = 0.049 \cdot L + 92.23$$

$$\sigma(L) = 0.016 \cdot L + 8.18$$

These expressions for $\mu(L)$ and $\sigma(L)$ are used in the Cumulative Distribution Function (CDF) of type I Extreme Value Distribution to calculate the (one-tailed) p-value of a given S^{AB} alignment score:

$$p(A,B) = \exp\left[-\exp\left(\frac{S^{AB} - \mu(L)}{\sigma(L)}\right)\right]$$

4. Similarity graphs and tables

Figure S4A shows the DFM similarity between the PDZ domains as a graph with the nodes representing the proteins and edges representing significant similarity. A similar graph can be made for the Z-scores obtained from Dali (Fig. S4B). We can plot the relationship to between these two scoring functions as shown in Fig. S5.

5. Correlation vs. DFM

To study the relationship between the dynamic fingerprint matrices and the correlation matrices commonly used in the analysis of proteins dynamics, we have plotted the two matrices against each other, calculated for the same MD simulation. That is, for each residue pair (i,j) the DFM value F_{ij} is assigned to the correlation value C_{ij}. For example, Fig S6 presents the graph for the 20 ns simulation of PSD-95 PDZ3. The number of points in the graph is the number of different residue pairs in the protein. Note that the correlation vs. DFM plot calculated for other MD simulations appears to be very similar to the example presented here.

For residue pairs of positive correlation, the graph shows a clear tendency between the correlation matrix and DFM entries: the higher the correlation, the lower the pairwise fluctuation (DFM entry). This tendency, however, is not a general rule even for residue pairs of positive correlation, as we see pairs of relatively high (>0.4) correlation and high (>0.07) fluctuation. Moreover, the inverse relationship between correlation and DFM values breaks

off at negative (<-0.1) correlations and high (>0.05) DFM values. In these regions of the graph, the two measures appear to be totally uncorrelated. It therefore follows that one cannot accurately predict the fluctuation of two residues which have negative correlation. Similarly, the correlation of two residues cannot be predicted if they have relatively high pairwise fluctuation. Very high (>0.1) pairwise fluctuation values, however, are typically found for pairs of negative correlation. It is important to note that the pairs of neighbouring (covalently bonded) amino acids form a distinct cluster with fluctuation values close to zero and positive (typically >0.6) correlation coefficients.

As a summary, we can say that although in a well-defined region of the graph there is a clear inverse tendency between the entries of the two matrices, in other regions they appear to be independent. Therefore we conclude that the dynamic fingerprint matrix and the correlation matrix provide two different measures for the characterization of protein dynamics.

6. Distribution of Fluctuations

We were also interested to know whether the residues that were identified as being dynamically similar were also residues that tended to exhibit small deviations. To answer this question we have looked at the DFM patterns at a more coarse-grained level. The DFM matrix values were discretized into three values: *low*, *medium* and *high* fluctuation. The thresholds of discretization were selected by analysing the total distribution of matrix entries collected from the 10 different DFMs corresponding to the studied PDZ domains (Fig. S7). The thresholds were set using the equal-frequency binning method; i.e. 1/3 of the values in this distribution were assigned to *low*, 1/3 of the values were assigned to *medium* and 1/3 of the values were assigned to *high* fluctuation.

Using this discretization scheme (thresholds calibrated for the total distribution of values), we can examine the frequency of *low*/*medium*/*high* fluctuation values in the collapsed DFMs identified by matrix alignment. For example, as discussed in the main manuscript, the dynamics-based alignment of PSD-95 PDZ3 and nNOS PDZ results in 77 x 77 collapsed DFM. As shown by a histogram in Fig. S8, the proportion of *low*, *medium* and *high* fluctuation values in the mean collapsed DFM pattern is 45 per cent, 40 per cent and 15 per cent, respectively. As we can see, a large proportion of the DFM entries included in the pattern indeed correspond to low pairwise fluctuations, but still a significant proportion of values correspond to medium and high fluctuations. Of course, the relative proportion of low/medium/high values may be different in the case of different protein pairs.

7. Additional Dynamic Profile

The dynamic profiles we obtain for a residue (such as Phe325 in the main manuscript) describe the motion of other residues with respect to that residue. As another example, we looked at Asp 348 which is located within helix 1. The profile is shown in Fig. S9 and is visually similar to that obtained for Phe325 (which is located at the end of a beta strand).

Supplementary Figures.

Figure S1. Shape of the logistic function.

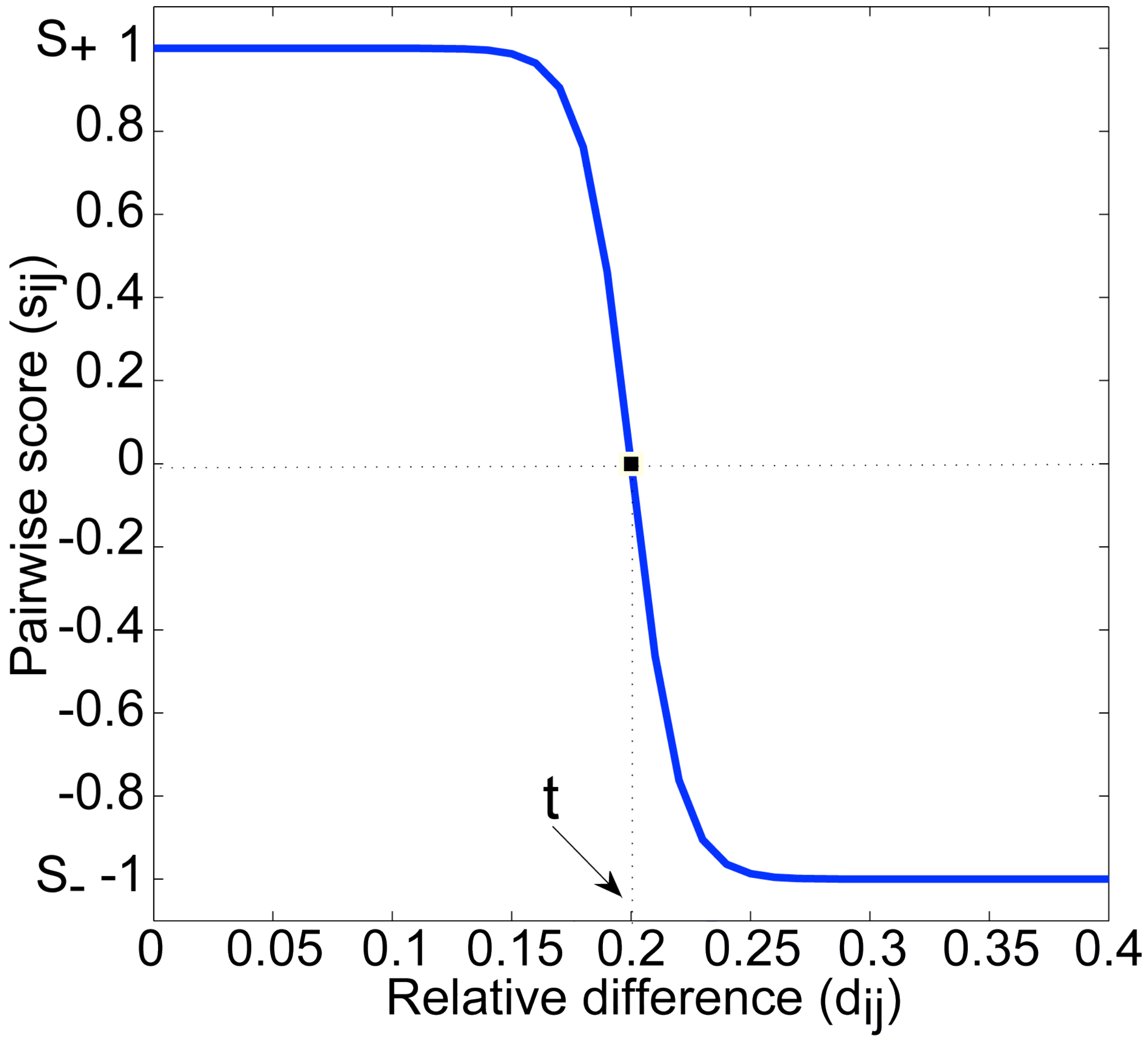

Figure S2. Example probability (extreme value) distribution of scores for pairs of proteins of length 80 residues.

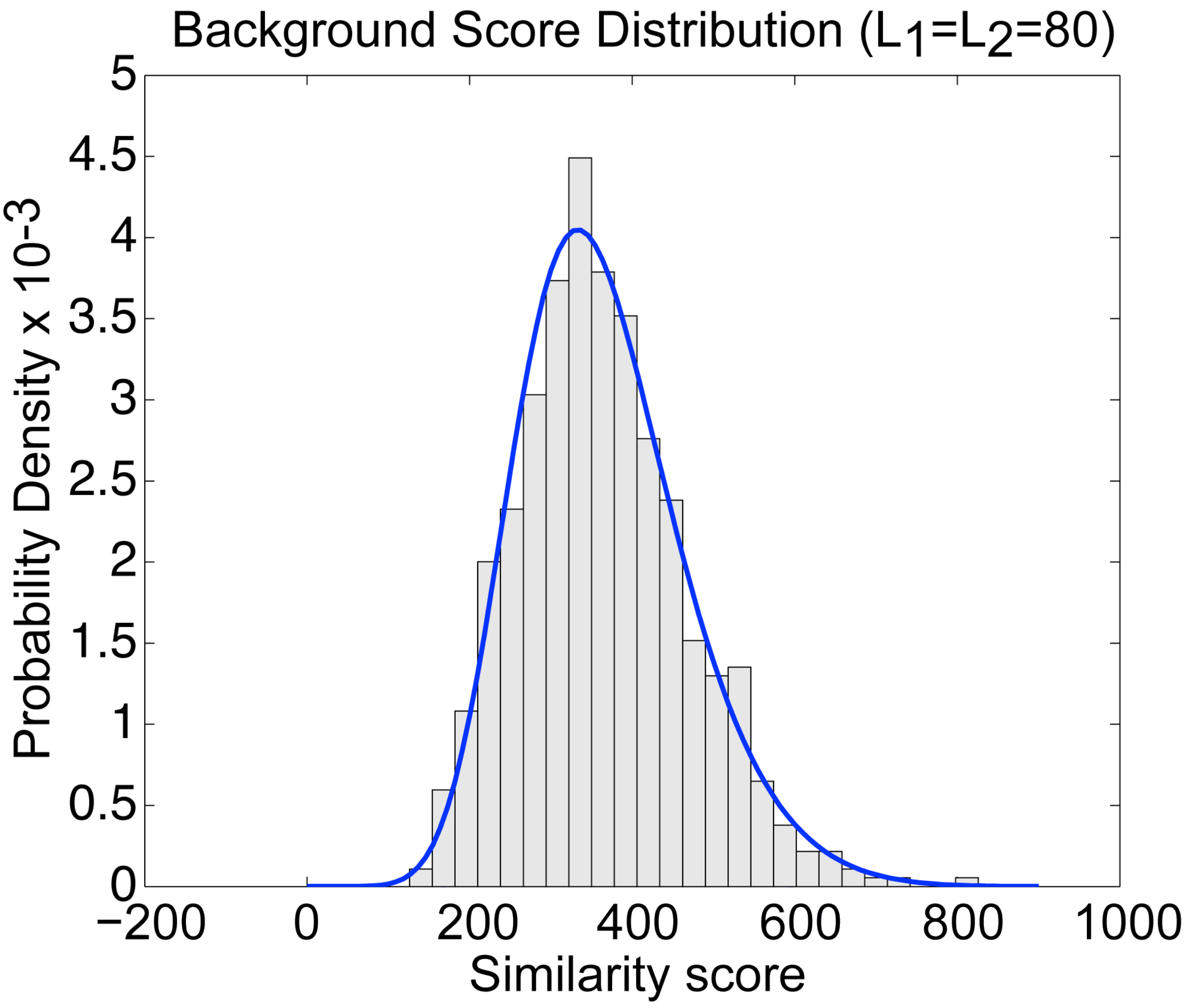

Figure S3. The dependence of the location and scale parameters on the L (the product of L1 and L2, where L1 is the length (in number of residues) of sequence 1 and L2 is length of sequence 2). See text for detailed description.

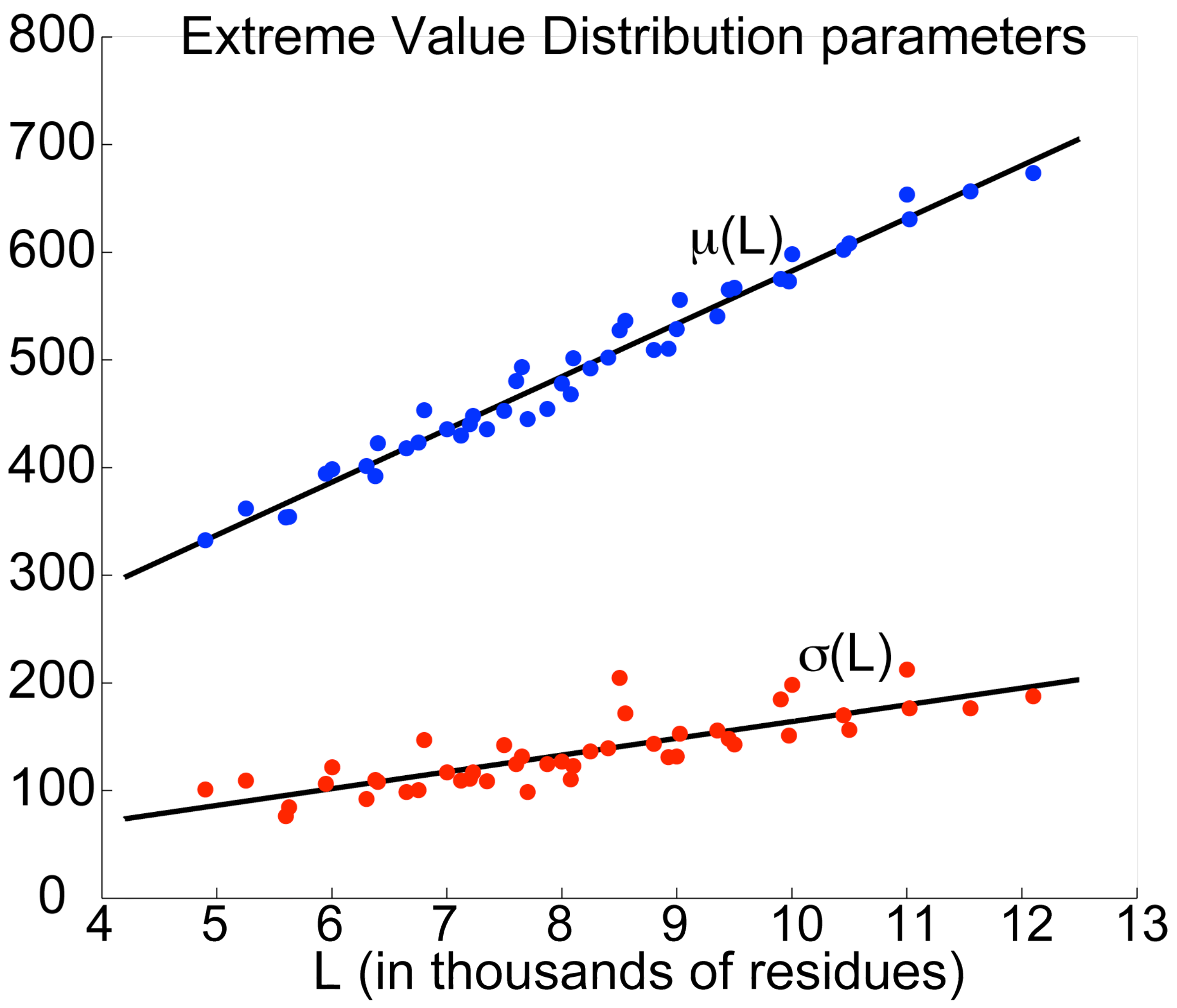

Figure S4. (A) Similarity graph of the 10 PDZ domains derived from DFM calculations. Nodes represent protein structures and edges represent $p > 0.05$. (B) Similarity graph calculated by Dali. Edges represent Z-scores > 8.5.

A

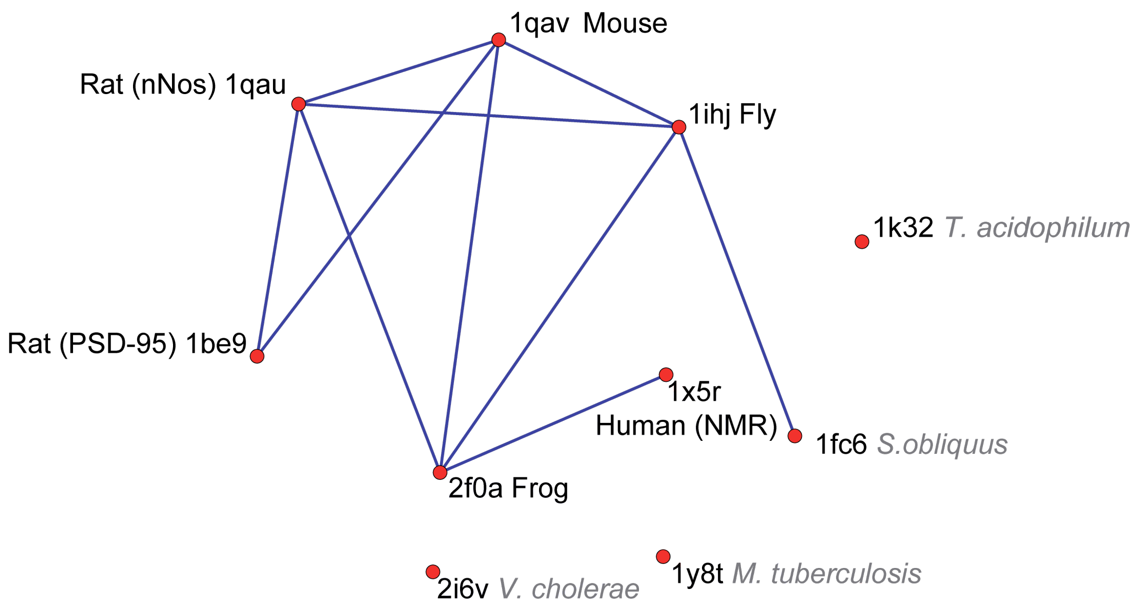

B

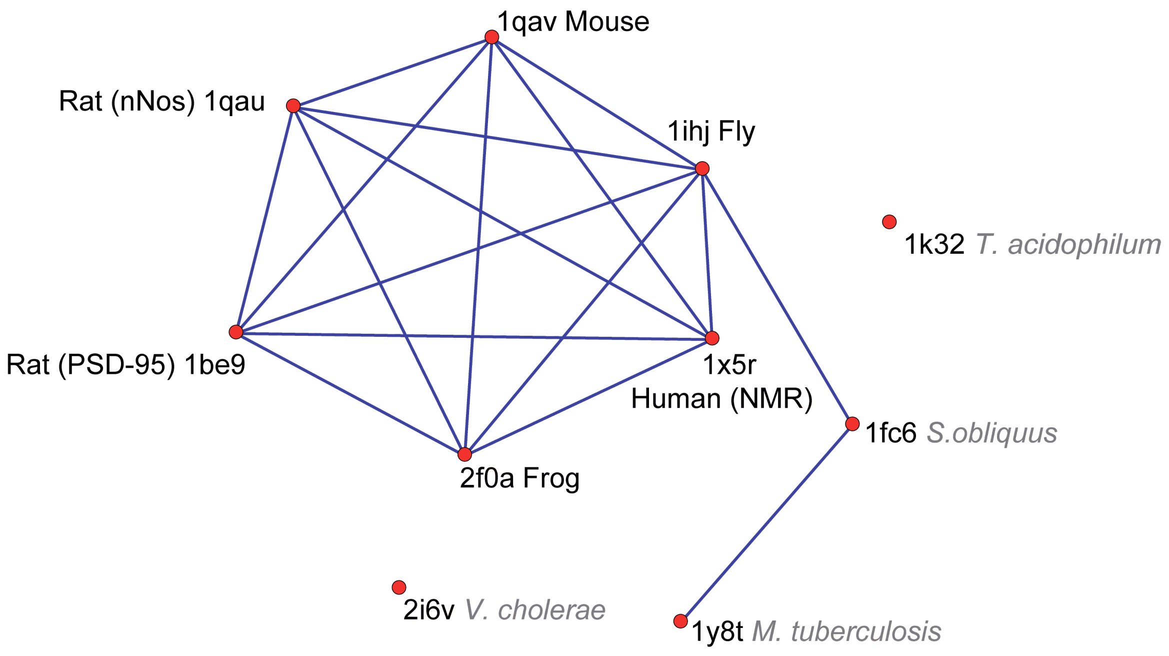

Figure S5. The correlation between the dynamics similarity (as computed by the DFM overlap) and the structural similarity as computed by Dali.

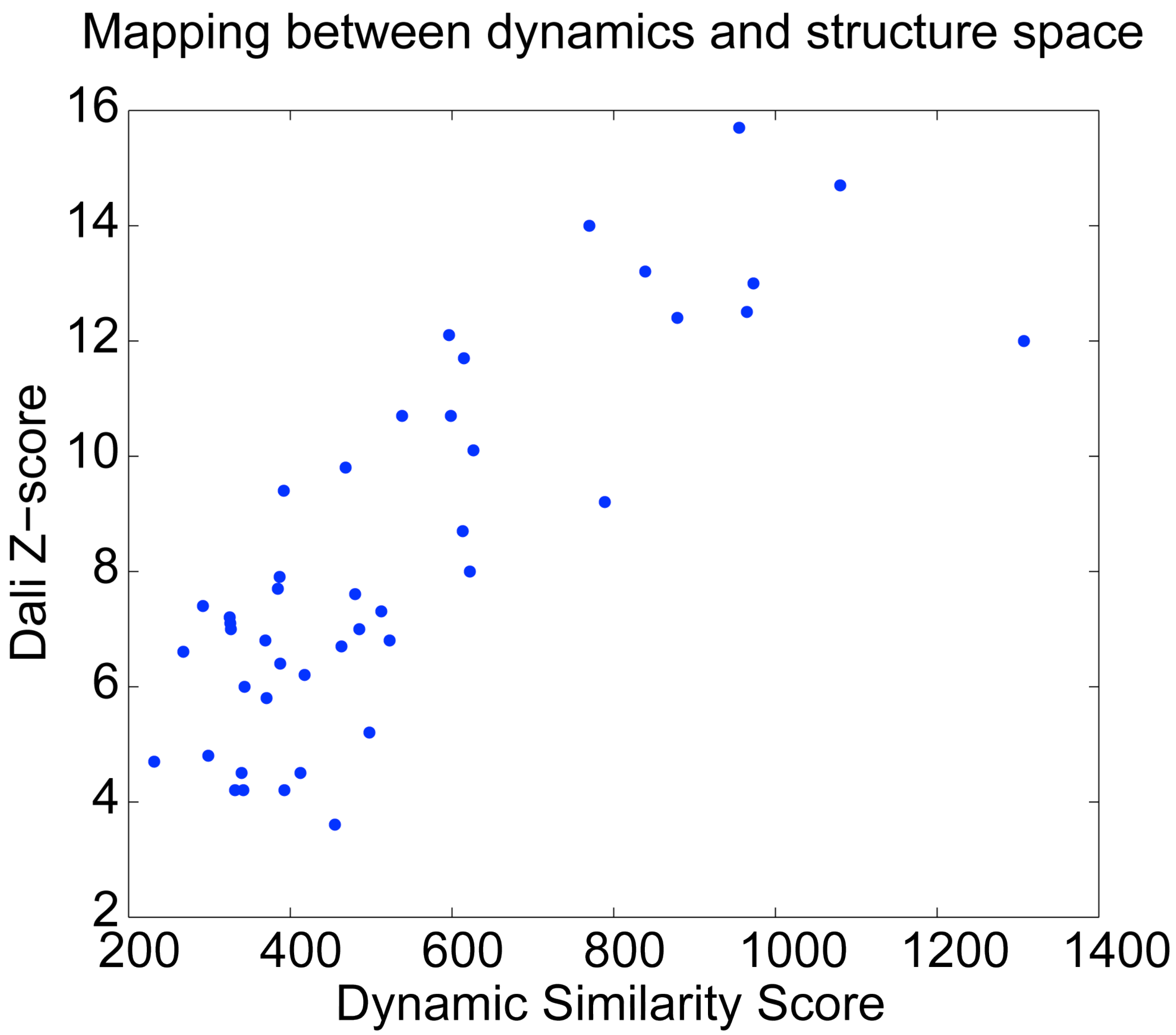

Figure S6. DFM versus correlation for all pairs of residues from a 20 ns simulation of PDZ3 of PSD-95.

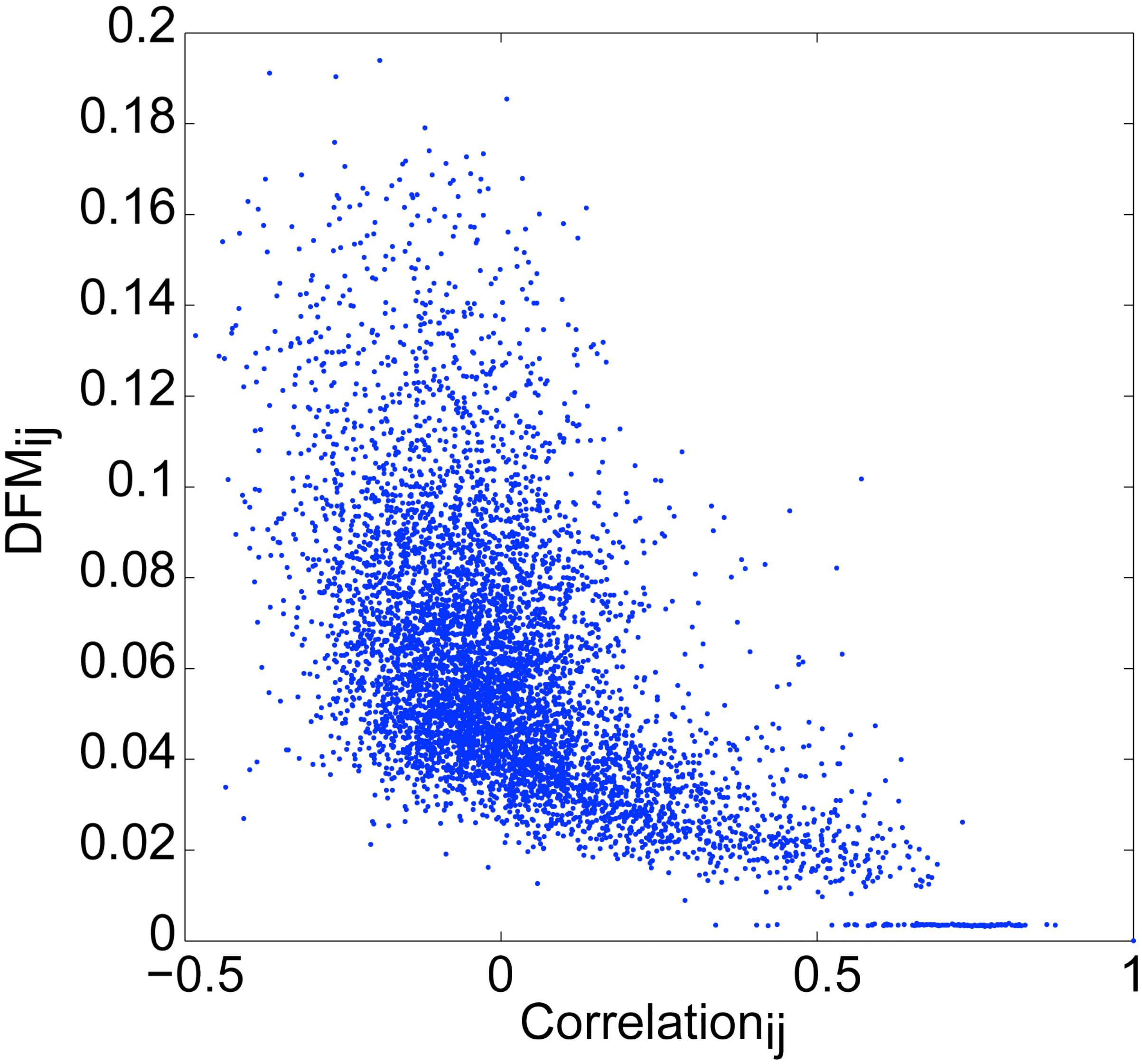

Figure S7. Histogram of DFM matrix entries from all 10 PDZ simulations.

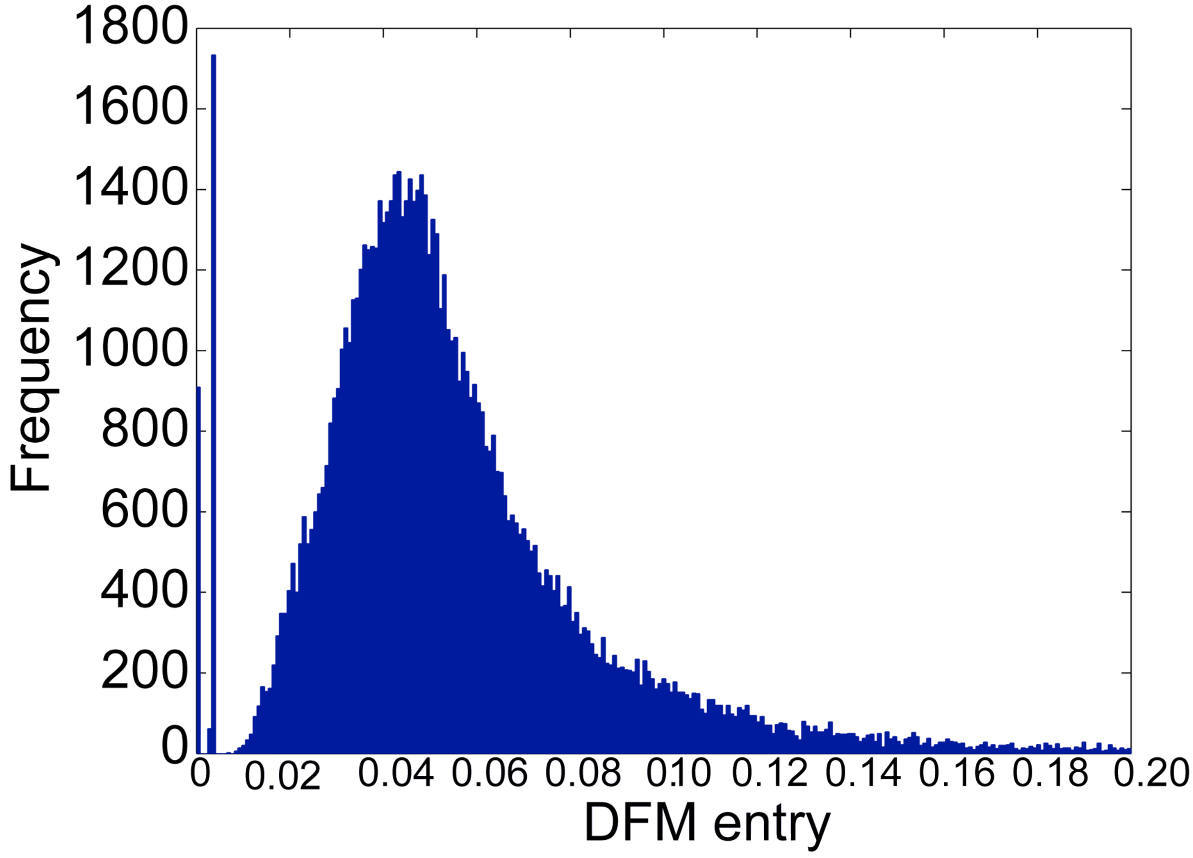

Figure S8. Categorization of fluctuations from the 20 ns simulation of 1be9 according to equal binning of the distribution shown in Figure S7.

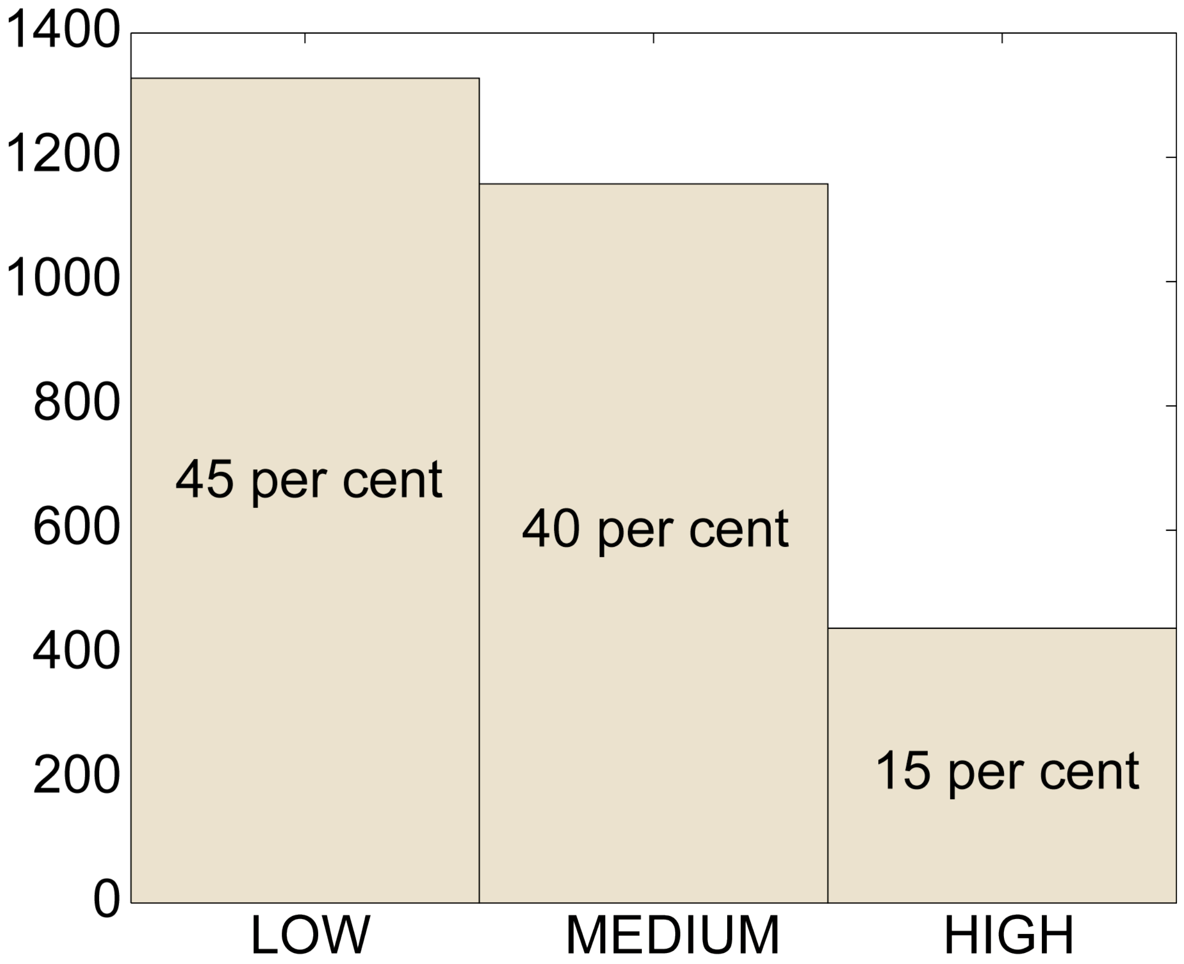

Figure S9. An example of a profile for a residue (Asp 348) situated within an α-helix.

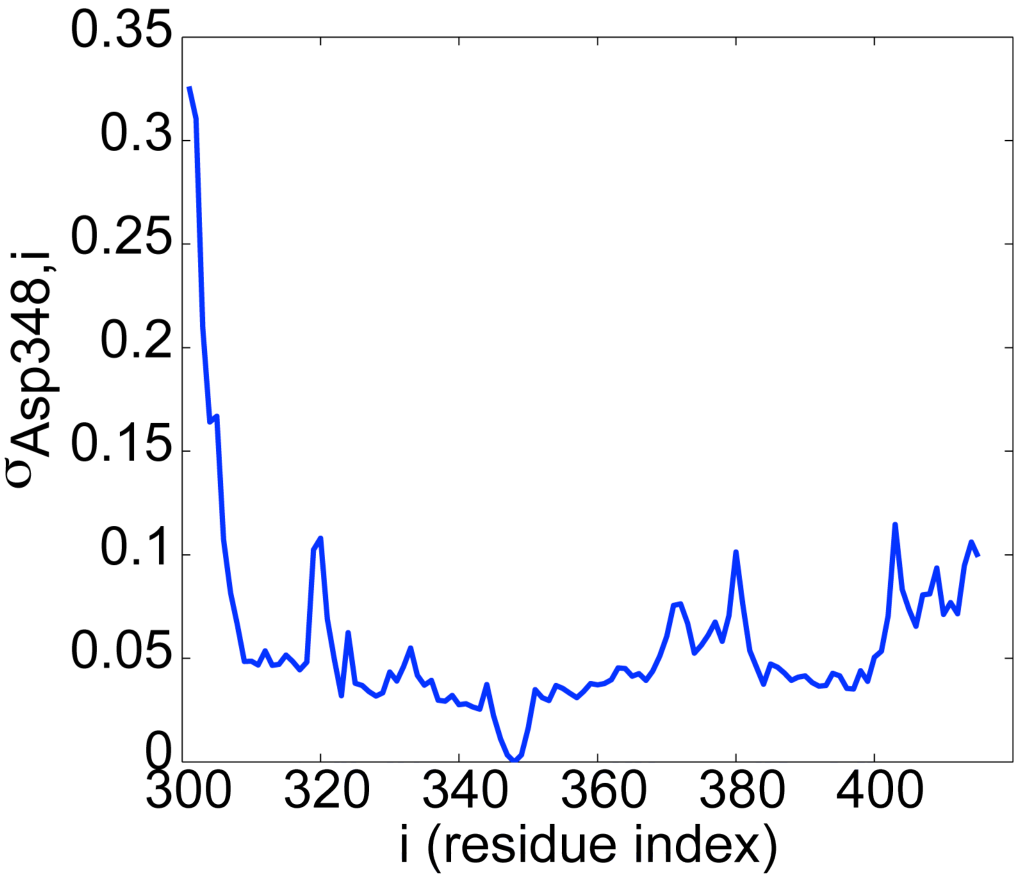

Supplementary Tables

Table S1. Dali Z-scores for the PDZ proteins considered.

	**1qau**	**1ihj**	**1be9**	**1k32**	**1x5r**	**1y8t**	**1fc6**	**1qav**	**2i6v**	**2f0a**
**1qau**										
**1ihj**	13.0									
**1be9**	12.0	12.1								
**1k32**	6.7	7.0	5.8							
**1x5r**	10.1	10.7	9.8	5.2						
**1y8t**	7.7	7.9	6.4	7.1	6.2					
**1fc6**	8.0	8.7	7.4	6.6	7.2	9.4				
**1qav**	14.7	14.0	15.7	6.8	10.7	7.3	7.6			
**2i6v**	4.5	4.8	3.6	6.0	4.2	4.5	4.7	4.2		
**2f0a**	12.5	12.4	11.7	6.8	9.2	7.0	7.9	13.2	4.2	

Table S2. Reference proteins used to derive the background score distributions.

Protein	PDB	Length	Source Organism	Resolution (Å)
Gamma subunit of the dissimilatory sulfite reductase (DsrC)	1SAU	114	*Archaeoglobus fulgidus*	1.12
S-adenosylmethionine decarboxylase (chain A)	1TLU	117	*Thermotoga maritima*	1.55
Origin binding domain of large T antigen (chain A)	2IPR	127	*Simian virus 40*	1.5
YueI protein (chain A)	2OHW	128	*Bacillus subtilis*	1.4
Lysozyme	1LZ1	130	*Homo sapiens*	1.5
Carbohydrate binding module (chain A)	1UXZ	131	*Cellvibrio mixtus*	1.4
Soluble Secreted Antigen MPT53	1LU4	134	*Mycobacterium tuberculosis*	1.12
Hypothetical protein Atu0741	1ZHV	134	*Agrobacterium tumefaciens str. c58*	1.5
Endonuclease V	2END	137	*Enterobacteria phage t4*	1.45
BclA protein	2R6Q	138	*Bacillus anthracis*	1.43
Cutinase	1AGY	197	*Nectria haematococca*	1.15
Antiviral protein DAP-30	1RL0	255	*Dianthus caryophyllus*	1.4
